# Heart rate variability parameters indicate altered autonomic tone in subjects with COVID-19

**DOI:** 10.1038/s41598-024-80918-w

**Published:** 2024-12-28

**Authors:** Gabriel Gruionu, Md Aktaruzzaman, Anita Gupta, Thomas V. Nowak, Matthew Ward, Thomas H. Everett

**Affiliations:** 1 Krannert Cardiovascular Research Center, Division of Cardiovascular Medicine, Department of Medicine, Indiana University School of Medicine, Indianapolis, IN USA; 2 Division of Gastroenterology-Hepatology, Department of Internal Medicine, Indiana University School of Medicine, Indianapolis, IN USA; 3 Weldon School of Biomedical Engineering, Purdue University, West Lafayette, IN USA

**Keywords:** Sympathetic nervous system, Parasympathetic nervous system, COVID-19, Heart rate variability, Translational research, Diagnostic markers, Predictive medicine, Biomedical engineering

## Abstract

COVID-19 is associated with long-term cardiovascular complications. Heart Rate Variability (HRV), a measure of sympathetic (SNS) and parasympathetic (PNS) control, has been shown to predict COVID-19 outcomes and correlate with disease progression but a comprehensive analysis that includes demographic influences has been lacking. The objective of this study was to determine the balance between SNS, PNS and heart rhythm regulation in hospitalized COVID-19 patients and compare it with similar measurements in healthy volunteers and individuals with cardiovascular diseases (CVD), while also investigating the effects of age, Body Mass Index (BMI), gender and race. Lead I ECG recordings were acquired from 50 COVID-19 patients, 31 healthy volunteers, and 51 individuals with cardiovascular diseases (CVD) without COVID-19. Fourteen HRV parameters were calculated, including time-domain, frequency-domain, nonlinear, and regularity metrics. The study population included a balanced demographic profile, with 55% of participants being under 65 years of age, 54% identifying as male, and 68% identifying as White. Among the COVID-19 patients, 52% had a BMI ≥ 30 compared to 29% of healthy volunteers and 33% of CVD patients. COVID-19 and CVD patients exhibited significantly reduced time-domain HRV parameters, including SDNN and RMSSD, compared to healthy volunteers (SDNN: 0.02 ± 0.02 s vs. 0.06 ± 0.03 s, *p* < 0.001; RMSSD: 0.02 ± 0.02 s vs. 0.05 ± 0.03 s, *p* = 0.08). In the frequency domain, both COVID-19 and CVD patients showed increased low-frequency (LF) power and lower high-frequency (HF) power compared to healthy volunteers (COVID-19 LF: 18.47 ± 18.18%, HF: 13.69 ± 25.80%; Healthy LF: 23.30 ± 11.79%, HF: 22.91 ± 21.86%, *p* < 0.01). The LF/HF ratio was similar in COVID-19 patients (1.038 ± 1.54) and healthy volunteers (1.03 ± 0.78). Nonlinear parameters such as SD1 were significantly lower in COVID-19 patients (0.04 ± 0.04 s vs. 0.08 ± 0.05 s, *p* < 0.01), indicating altered autonomic regulation. Variations in HRV were observed based on demographic factors, with younger patients, females, and non-white individuals showing more pronounced autonomic dysfunction. COVID-19 patients exhibit significant alterations in HRV, indicating autonomic dysfunction, characterized by decreased vagal tone and sympathetic dominance, similar to patients with severe cardiovascular comorbidities. Despite higher heart rates, the HRV analysis suggests COVID-19 is associated with substantial disruption in autonomic regulation, particularly in patients with specific demographic risk factors.

## Introduction

COVID-19, caused by the SARS-CoV-2 virus infection, has been shown to impact multiple organ systems, including the cardiovascular system, leading to complications such as myocarditis, hypertension, arrhythmias, and autonomic dysfunction^1–4^. Autonomic dysfunction, which can present as palpitations, tachycardia with mild activity, hypo- or hypertension, and peripheral vasoconstriction, is particularly concerning in patients with underlying cardiovascular conditions, such as hypertension, coronary artery disease, arrhythmias, and cardiomyopathy, as these patients are at higher risk of severe outcomes^3^.

Heart rate variability (HRV) is a non-invasive, indirect measure of the balance between sympathetic and parasympathetic activity of the autonomic nervous system (ANS) function^3,5,6^. While HRV has been used in various clinical contexts, research examining HRV alterations in COVID-19 patients, particularly in relation to demographic and clinical variables, remains limited. Emerging evidence suggests that HRV can serve as an indicator of disease severity and the extent of autonomic dysfunction even in long-COVID-19 patients^7^. Common HRV metrics, including time-domain, frequency-domain, and nonlinear measures, offer a comprehensive way to assess autonomic regulation over both short-term (5-minute) and long-term (24-hour) ECG recordings^1,5^.

Several studies have demonstrated the potential of HRV parameters to identify and predict COVID-19 infection and related symptoms. Significant changes in HRV, such as reductions in the standard deviation of the inter-beat interval of normal sinus beats (SDNN), have been observed even before a COVID-19 diagnosis, highlighting the sensitivity of HRV as an early indicator of autonomic imbalance^3^. Other research has shown that COVID-19 patients with chronic atrial fibrillation (cAF) experience further reductions in HRV compared to pre-infection levels, even at similar average heart rates, with a pronounced decrease in vagal tone and poorer clinical outcomes^8^. Specifically, patients with severely depressed HRV, as indicated by low pNN50% values, faced higher mortality risks compared to those with preserved HRV^6^.

Age is a well-established factor influencing COVID-19 outcomes, with older individuals facing worse prognoses^4,9,10^. A higher SDNN values in older patients correlate with better survival rates, while lower SDNN values predict ICU admission^11^. A pilot study reported a median age of 62 years among survivors compared to 71 years for non-survivors of critical COVID-19, underscoring the impact of age on autonomic dysfunction and outcomes^12^. Similarly, median ages of 68 years for survivors and 71 years for non-survivors of COVID-19 are reported^11^.

Our study utilized a comprehensive set of heart rate variability (HRV) metrics to assess autonomic function in three distinct groups: (1) patients hospitalized with acute SARS-CoV-2 infection, (2) patients hospitalized with cardiovascular disease (CVD), and (3) healthy volunteers. We aimed to determine if COVID-19 infection leads to significant autonomic dysfunction, specifically hypothesizing that these patients would show reduced parasympathetic activity and increased sympathetic modulation. Recognizing the potential influence of demographic factors such as age, race, gender, and BMI on autonomic nervous system (ANS) function, we also examined how these variables might affect the degree of dysregulation in COVID-19 patients. Given that the healthy volunteer group was generally younger than both the CVD and COVID-19 groups, we stratified participants into two age categories (under and over 66 years) to account for age-related autonomic differences. Through detailed intra-group and inter-group HRV comparisons, our goal was to clarify how demographic characteristics modulate autonomic responses in the context of COVID-19 and to highlight any patterns that could inform personalized treatment strategies.

## Methods

### Study population and clinical characteristics

The study was approved by the Indiana University Institutional Review Board, and all research was performed in accordance with all relevant guidelines and regulations and in accordance with the Declaration of Helsinki. All patients provided informed consent for the Lead I ECG recordings. COVID-19 and CVD control patients were recruited from the Cardiology Division of the Indiana University Health Methodist Hospital between September 2020 and September 2021 (Table 1), and CVD control patients had tested negative for COVID-19.

### Patient selection process

Inclusion criteria for the COVID-19 and CVD groups required participants to be over 18 years, not pregnant, and not dependent on a pacemaker. Exclusion criteria included a history of neurocardiogenic syncope, significant arrhythmias, or active myocarditis. For healthy volunteers, eligibility criteria required participants to be over 18, have no history of COVID-19 or cardiovascular diseases, and be free from arrhythmias or comorbidities affecting the autonomic nervous system. Volunteers were also screened for medication or substance use that could influence autonomic function. The sample size used in this study includes all patients available during this period who met the inclusion/exclusion criteria, reflecting the unique and temporary circumstances of the COVID-19 pandemic.

### ECG acquisition process

1. **Healthy Volunteers**: ECG recordings were obtained in a controlled environment using customized high-fidelity ECG equipment (10 kHz sampling frequency). Participants lay supine in a quiet room set at 70 °F, and recordings lasted 20 min. To minimize artifacts, participants were instructed to remain still and silent, with a trained operator monitoring for any movements or disturbances.
2. **COVID-19 and CVD Patients**: ECGs were collected via hospital telemetry systems (240/250 Hz) as part of standard care, with recordings lasting 15–60 min. To standardize data, segments with significant movement or clinical interventions were excluded, ensuring only consistent signal quality was analyzed.

The study population consisted of 31 healthy volunteers (mean age 33 ± 15 years, BMI 28 ± 6), 50 COVID-19 patients (mean age 63 ± 18.2 years, BMI 30.6 ± 6.8), and 51 CVD patients (mean age 71.2 ± 11 years, BMI 28.4 ± 5.2). p-values and effect sizes were calculated (Table 1), and patients were further segmented by gender, age, race, and BMI for analysis. The COVID group had cardiovascular complications and comorbidities similar to the CVD group, including arrhythmias, coronary artery disease, heart failure, and gastroesophageal reflux disease (Table 1).

**Table 1:** Demographic categories and clinically reported comorbidities for the study populations. The study population was segmented for age (≤ 65 adult, > 65 older adults), BMI (body Mass Index, < 30 normal or overweight, ≥ 30 obese), gender (male vs. female) and race (white vs. non-white). * 19 test participants (3 from COVID, 9 CVD and 7 healthy) had BMI data missing. CAD - coronary artery disease, CKD - chronic kidney Disease, COPD - Chronic Obstructive Pulmonary Disease. - no comparison test was performed.

Demographic Categories Number % of total		Total	COVID-19	Healthy volunteers	CVD control	*p*-values, effect size		
		132 100%	50 38%	31 23%	51 39%	COVID vs. CVD	COVID vs. Healthy	CVD vs. Healthy
Age	≤ 65yrs	73 55%	24 48%	29 94%	20 39%	0.64, 0.16	< 0.05, 0.63	< 0.05, 0.73
Gender	Male	71 54%	27 54%	15 48%	29 57%	0.77, 0.03	0.62, 0.06	0.46, 0.09
Race	White	90 68%	41 82%	19 61%	30 59%	0.02, 0.25	0.04, 0.23	0.83, 0.03
BMI*	< 30	61 46%	21 42%	15 48%	25 49%	0.22, 0.21	0.09, 0.27	0.44, 0.20
Clinically Reported Comorbidities								
Obesity		13 10%	8 16%	0	5 10%	0.37, 0.09	-	-
Hypertension		77 55%	35 68%	0	42 78%	0.10, 0.17	-	-
Chronic heart failure		17 12%	7 14%	0	10 18%	0.42, 0.08	-	-
Atrial fibrillation		18 14%	5 10%	0	13 25%	0.04, 0.21	-	-
Diabetes		25 11%	9 18%	0	16 31%	0.11, 0.16	-	-
CAD		32 24%	12 24%	0	20 39%	0.09, 0.17	-	-
CKD		18 14%	8 16%	0	10 20%	0.60, 0.05	-	-
COPD		10 8%	3 6%	0	7 14%	0.18, 0.13	-	-

### Data analysis

**Fig. 1:**
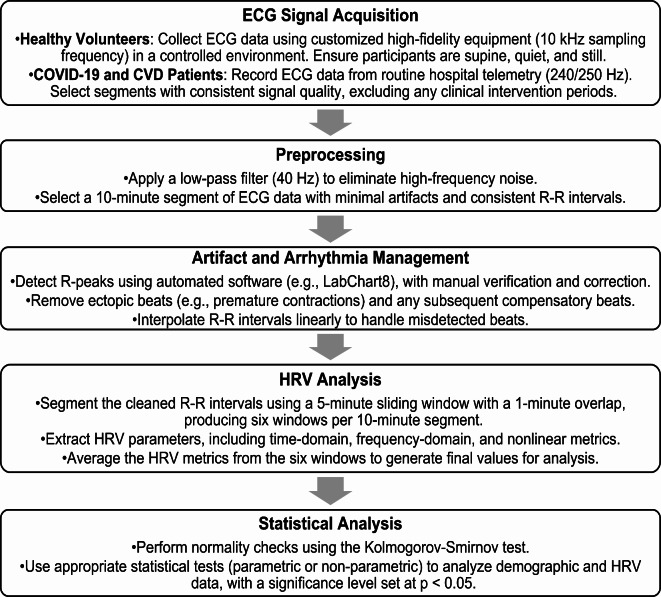
Workflow for HRV analysis. The data analysis starts with acquisition of Lead I ECG telemetry recording, preprocessing, R-wave peak detection, and R-R interval segmentation with overlapping windows. Fourteen HRV parameters are subsequently extracted to assess heart rate variability.

A structured workflow was followed for HRV analysis (Fig. 1).

The ECG data were saved as ASCII text files and analyzed using custom software written in MATLAB R2022b (The MathWorks, Inc., Natick, Massachusetts, USA). The digitized ECG signals were low-pass filtered at 40 Hz to remove high-frequency noise, and a 10-minute segment of each recording, containing minimal motion artifacts, 60 Hz noise, or arrhythmias, was selected for analysis (Fig. 1)^13^. R-peaks were detected using the ECG analysis module of LabChart8 (AD Instruments Inc., Colorado Springs, CO, USA), and any missed beats or arrhythmias were manually corrected (Table 2). Ectopic beats, such as premature atrial or ventricular contractions, along with the subsequent compensatory beat, were removed. Misdetected beats due to artifacts were also eliminated, and the corresponding R-R intervals were estimated via linear interpolation^13^. Short-term HRV analysis was performed using a 5-minute sliding window with 1-minute overlap, yielding 6 data points from each 10-minute recording. After processing the R-R series (Fig. 1), a set of 14 HRV parameters were extracted from each analysis window. The average estimate of the HRV parameters of the 6 windows were considered as the HRV parameters for the RR series.

### HRV parameters analyzed

The 14 HRV parameters that were extracted included time domain, frequency domain, nonlinear, and regularity (complexity) analysis of the RR series as described below:

### Time domain parameters

MeanNN [s]: The estimated mean values of the RR series, measured in s.

SDNN [s]: The standard deviation of all RR intervals in the series, measured in s.

RMSSD [s]: The square root of the mean of sum of squares of differences between the adjacent RR intervals, measured in s.

pNN50 [%]: The percentage of number of adjacent pairs of RR intervals which differs by more than 50 ms in the RR series.

#### Frequency domain parameters

In the frequency-domain, we considered (%) of power in three frequency bands, very low frequency (VLF): [0.003 0.04] Hz, low frequency (LF): [0.04 0.15] Hz, and high frequency (HF): [0.15 0. 4] Hz expressed in normalized units, which represents the relative value of each power component in proportion to the total power minus the VLF component to emphasize the behavior of the autonomic nervous system^14^.

VLF [%]: The power [%] in the very low frequency band (0.003 0.04) Hz was estimated by taking the percentage of normalized power in VLF band to the total power of the RR series.

LF [%]: The power [%] in low frequency band (0.04 0.15) Hz was estimated by taking the percentage of normalized power in LF band to the total power of the series.

HF [%]: The power [%] in high frequency band (0.15 0.4) Hz was estimated by taking the percentage of normalized power in HF band to the total power of the series.

LF/HF: The ratio of low frequency power (LF) to the HF.

### Nonlinear measures

For non-linear analysis, Poincaré plots were constructed, and an ellipse was fitted with 90% confidence around the distribution of the data. The lengths of semi-minor (SD1) and semi-major (SD2) axis of the ellipse, and the ratio SD1/SD2 and SD2/SD1 were determined^15,16^.

### Regularity measures

Sample entropy (SampEn)^14^ was used to measure the regularity (or complexity) of the R-R series as: $\:SampEn\left(m,r,N\right)=\text{log}{P}_{m}-\text{log}{P}_{m+1}$, where *P*_*m*_ and *P*_*m+1*_, are the probability of matching templates of length *m* and an extended *m* + 1 within tolerance *r*. Two vectors are considered similar if the maximum absolute difference between the corresponding elements of them is *r*.

The probability of agreement (probAgr) was used as a parametric approach to provides more insights about the nonlinearity, non-stationarity and / or non-Gaussian presence in the series^17^.

### Statistical analysis

Demographic data were presented as percentages or mean ± standard deviation, while HRV parameters were reported as median ± interquartile range (IQR) due to their skewed distribution. Continuous variables (age and BMI) were analyzed using the Wilcoxon Rank Sum test, and categorical variables (gender and race) with the Chi-Square test (Table 1). The Kolmogorov-Smirnov test assessed normality of HRV parameters. Depending on data distribution, we applied either the Student’s t-test or Wilcoxon Rank Sum test. To control for type-1 errors, we used one-way ANOVA or the Kruskal-Wallis test. Statistical significance was set at *p* < 0.05.

## Results

### Demographic categories and clinically reported comorbidities for study populations

The study participants show distinct differences in demographics and comorbidities across the COVID-19, healthy volunteer, and cardiovascular disease (CVD) groups (Table 1). Older age and higher BMI are more prevalent in both the COVID-19 and CVD groups compared to the healthy population, with obesity significantly more common in these groups. Comorbidity conditions such as hypertension, chronic heart failure, and coronary artery disease are predominantly seen in the CVD group, with clear differences in their frequency compared to the other groups. Additionally, chronic kidney disease (CKD) and chronic obstructive pulmonary disease (COPD) are more frequent in the COVID-19 and CVD groups, while these conditions are rare in the healthy population. These variations underline the differing health profiles of each group, particularly the elevated cardiovascular and metabolic risk factors in both COVID-19 and CVD patients. Gender distribution is also noted, with a slight male predominance in both the COVID-19 and CVD groups, though this was not statistically significant. The race distribution reflects a higher proportion of white participants across all groups, with no significant racial differences noted between the COVID-19 and CVD groups.

The number of patients with detected arrhythmias on the recordings are shown in Table 2. The two types of arrhythmias observed in all patient groups during the entire recording period, and specifically during a 10-minute segment were atrial fibrillation (Afib) and premature atrial or ventricular contractions (pACs/pVCs) (Table 2). The CVD group had more occurrences of arrhythmias than COVID-19, and healthy individuals (Table 2).

**Table 2:** Detected arrhythmias, including atrial fibrillation (Afib) and premature atrial or ventricular contractions (pACs/pVCs), in COVID-19, CVD control, and healthy volunteer groups.

Patient Group	No. of cases with arrhythmia during the entire recording		No. of cases with arrhythmia during the 10-min segment	
	Afib	pACs/ pVCs	Afib	pACs/pVCs
COVID-19	5	8	3	6
CVD Control	7	8	4	6
Healthy Volunteers	None	1	None	1

### Comparison of HRV parameters across groups

**Fig. 2:**
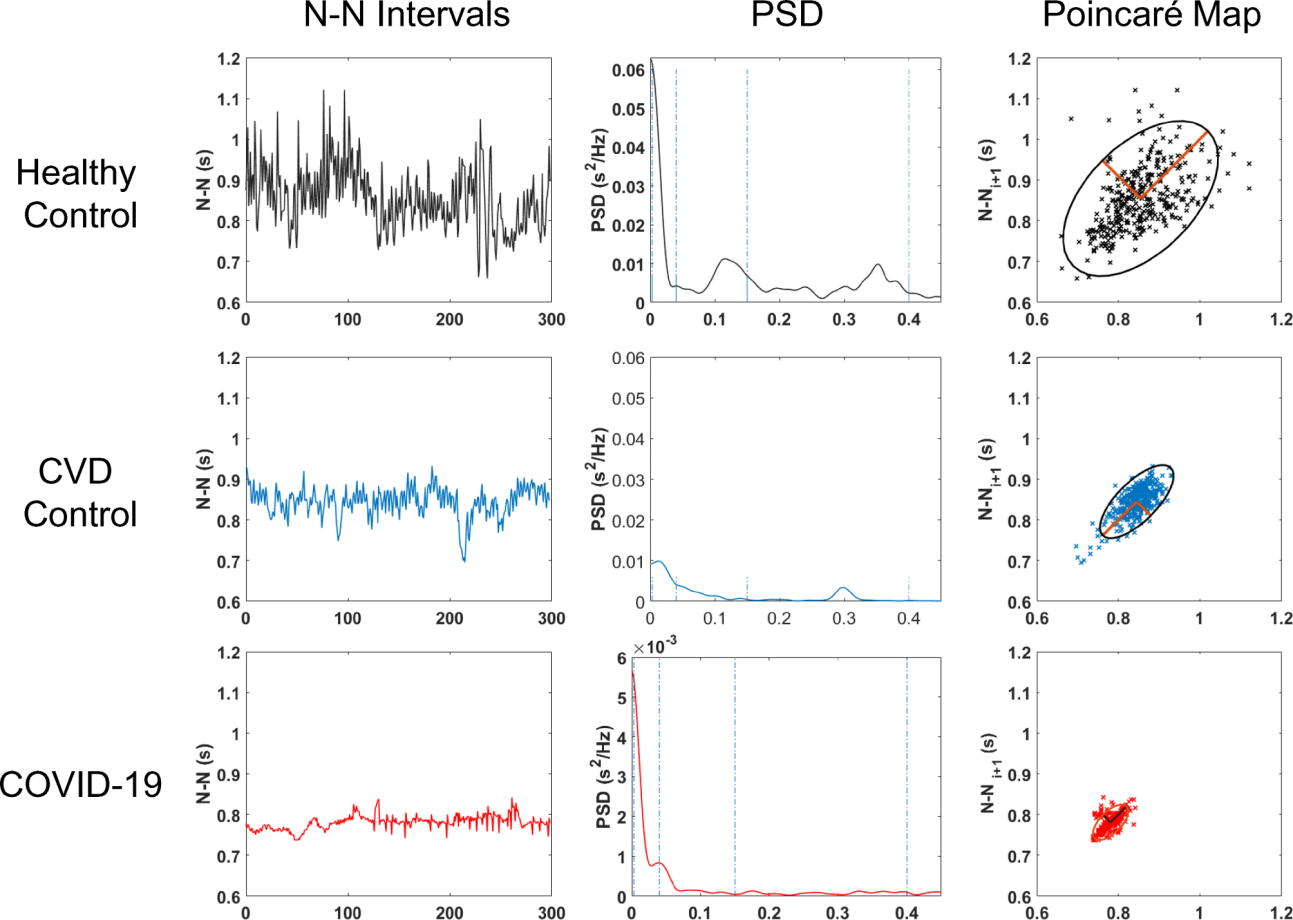
Linear and nonlinear HRV parameters for a sample RR series from each group. The rows in panels (**a**), (**b**), and (**c**) represent the HRV parameters extracted from the RR series of subjects from the groups: healthy control, CVD control, and COVID-19 patients, respectively. The first column displays the time-series of NN intervals (in seconds), the second column presents the power spectral density (PSD) analysis, and the third column shows the Poincaré plots depicting the relationship between successive NN intervals (NN_n vs. NN_n + 1). The healthy volunteers typically exhibited higher variability compared to the CVD and COVID-19 groups.

The analysis of multiple HRV parameters revealed several differences between the groups as illustrated for a representative sample for each experimental group (Fig. 2). The time-series analysis of the NN intervals shows higher variability in healthy volunteers compared to the more stable patterns observed in the CVD and COVID-19 groups. This variability is further reflected in the power spectral density (PSD) plots, where healthy volunteers exhibit more prominent low- and high-frequency components, indicating a higher degree of autonomic regulation. In contrast, the CVD and COVID-19 groups show reduced spectral power in these frequency ranges, with a shift towards lower overall variability. The Poincaré plots show the increased dispersion in healthy volunteers, consistent with greater HRV, while the CVD and COVID-19 groups display more condensed clusters, suggesting diminished HRV.

**Fig. 3:**
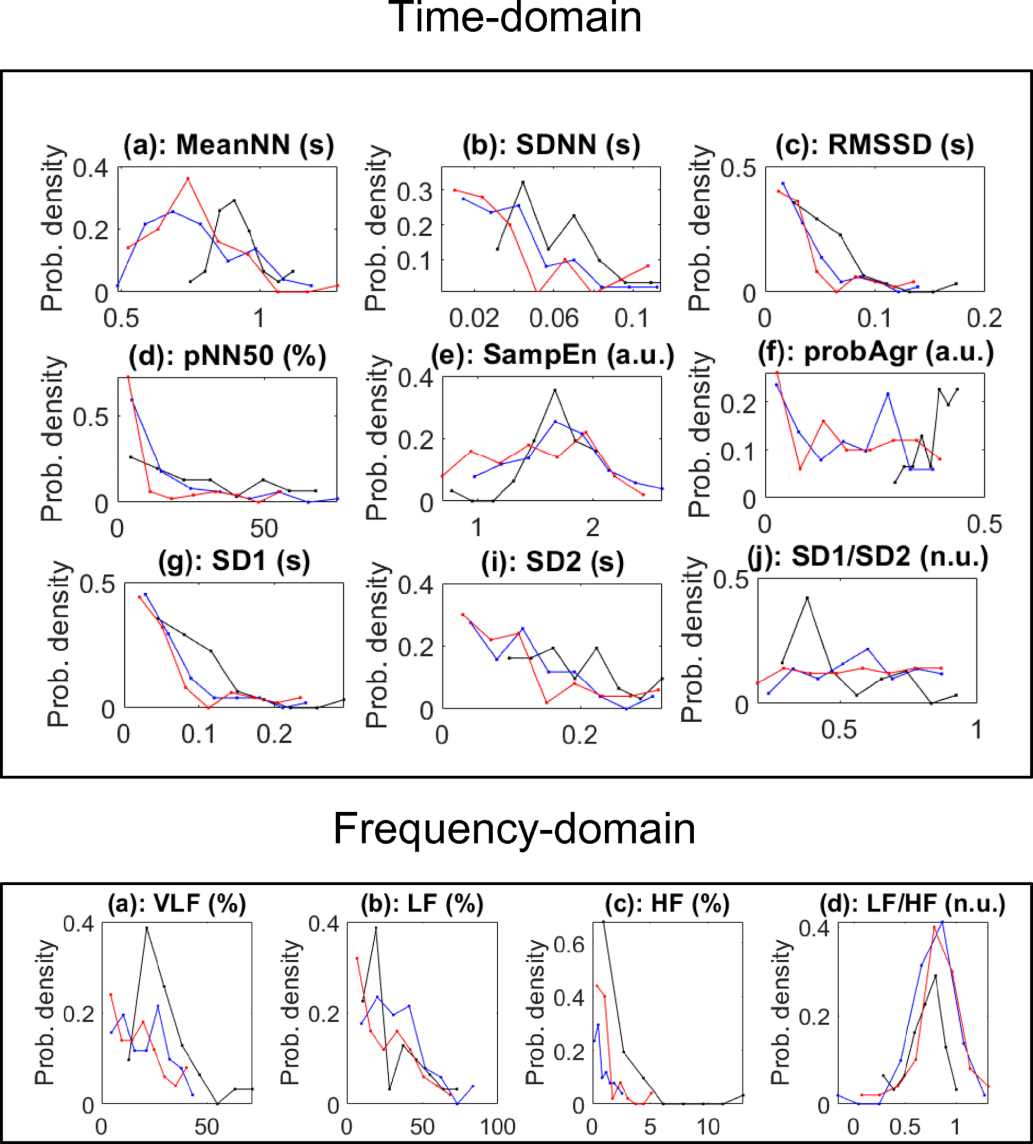
The probability distribution of HRV parameters. The graphs lines represent the distributions for COVID-19 (red lines), healthy volunteers (black lines), and CVD control (blue lines) for time (a-i) and frequency (j-m) domains. X-axis represents the parameter values, while Y-axis represents probability density.

To understand the global differences in autonomic function between groups, we co-displayed the probability distribution of HRV parameters for the three patient cohorts for the time (Fig. 3, top panels) and frequency-domains (Fig. 3 bottom panels). For the time-domain parameters, MeanNN showed a notable shift between the groups, with healthy volunteers displaying a wider distribution compared to CVD and COVID-19 patients (Fig. 3a, top). The lowest value of the MeanNN was 0.72 ± 0.16 s and was found in COVID-19 whereas the highest 0.917 ± 0.099 s was in the healthy volunteers. SDNN showed lower variability in both COVID-19 and CVD groups compared to the healthy volunteers, indicating reduced overall HRV (Fig. 3b). RMSSD exhibited a similar trend, with reduced parasympathetic activity in COVID-19 and CVD patients relative to the healthy controls who had a significantly higher RMSSD (0.05 ± 0.03 s**)** than other groups (COVID-19: 0.02 ± 0.02 s, and CVD control: 0.03 ± 0.02 s). (Fig. 3c). pNN50 was significantly lower in the COVID-19 group, suggesting impaired parasympathetic regulation (Fig. 3d). The healthy volunteers had a pNN50% of 20.99 ± 38.30 which was higher than other groups (COVID-19: 0.79 ± 11.75, and CVD control: 5.98 ± 17.02). SampEn values were lower in the COVID-19 and CVD groups, reflecting reduced HRV complexity (Fig. 3f). ProbAgr showed a distinct separation between groups, with the healthy volunteer group showing higher density values (Fig. 3g). Both COVID-19 and CVD patients show lower SD1 values compared to healthy volunteers, indicating a reduction in short-term HRV and parasympathetic activity in these populations (Fig. 3h). SD2 probability density curves for COVID-19 and CVD patients are also shifted downward compared to healthy volunteers, suggesting reduced overall long-term variability from a combination of both sympathetic and parasympathetic influences in these patients, which is characteristic of autonomic dysfunction (Fig. 3i). COVID-19 and CVD patients exhibit altered SD1/SD2 ratios compared to healthy volunteers, further supporting the presence of autonomic imbalance, with a reduced parasympathetic contribution in these populations (Fig. 3i).

In the frequency-domain, the VLF power was markedly higher in COVID-19 patients, compared to healthy volunteers and CVD controls (Fig. 3j). LF was elevated in the COVID-19 group, indicating altered autonomic sympathetic and parasympathetic activity balance (Fig. 3k). High-frequency power, HF, which reflects parasympathetic activity, was significantly reduced in the COVID-19 and CVD groups (Fig. 3l). LF/HF, a marker of sympatho-vagal balance, was increased in COVID-19 and CVD patients compared to the healthy control group, suggesting a shift toward sympathetic control (Fig. 3m). Overall probability distribution data show that both COVID-19 and CVD patients exhibit significant reductions in HRV parameters, with COVID-19 patients showing a shift toward sympathetic control and impaired parasympathetic regulation compared to healthy controls. Moreover, the SD1, SD2, and SD1/SD2 values reveal that both COVID-19 and CVD patients exhibit significant reductions in short-term and long-term HRV, as well as an altered balance between these components, indicating overall autonomic dysfunction, particularly in parasympathetic regulation.

### Correlation between time, frequency, and nonlinear HRV parameters

**Fig. 4:**
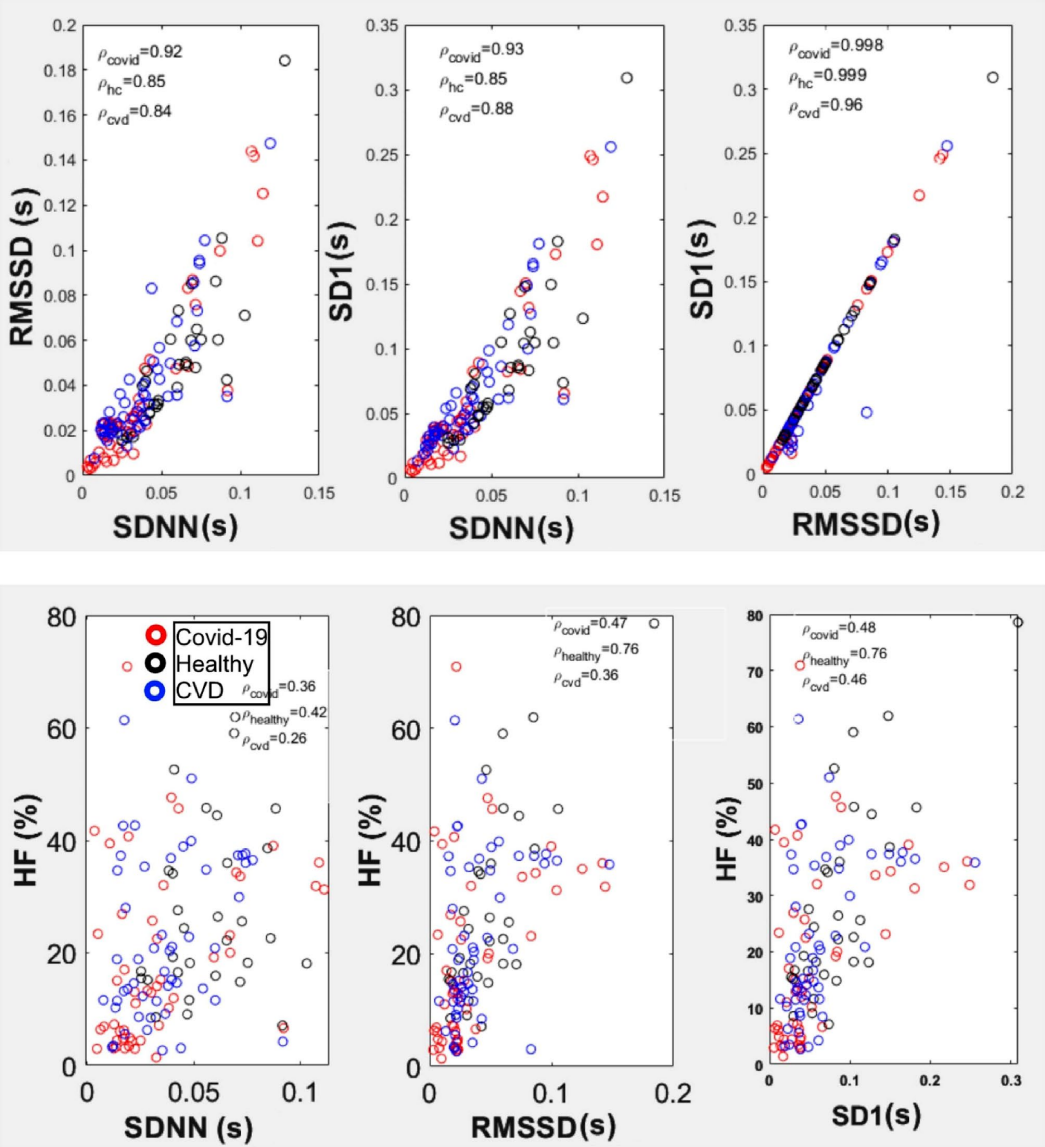
Correlation between time-domain parameters (SDNN, RMSSD) and the nonlinear parameter (SD1) (top pannels) and correlation between high-frequency (HF) power (%) and time-domain/nonlinear parameters (SDNN, RMSSD, SD1) (bottom panels) for COVID-19 patients (red), healthy volunteers (blue), and CVD control patients (black). The correlation coefficients, ρ -values are displayed for each subject group: COVID-19 (ρ_covid_), healthy volunteers (ρ_healthy_), and CVD control (ρ_cvd_).

To gain deeper insight into the relationship between time-domain, frequency-domain, and nonlinear HRV parameters, we examined the correlations between these metrics across the three subject groups (Fig. 4). The correlations between key time-domain and nonlinear HRV parameters: SDNN, RMSSD, and SD1 show strong correlations between SDNN and RMSSD, SDNN and SD1, and RMSSD and SD1, as reflected by the high correlation coefficients (ρ -values) displayed for each group. These strong relationships highlight the interconnectedness of time-domain and nonlinear parameters, all of which reflect short-term HRV and parasympathetic regulation. Similarly, the correlations between the high-frequency (HF) component of the frequency domain and the same key time-domain and nonlinear HRV parameters (SDNN, RMSSD, SD1) demonstrate the strength of the association between HF and these parameters across. The strong correlations between HF and time-domain measures suggest that changes in parasympathetic activity (HF) are closely related to alterations in HRV metrics (SDNN, RMSSD, SD1), which are critical for autonomic regulation.

The trends observed above were supported by One-Way ANOVA analysis of HRV parameters across time, frequency, regularity, and nonlinear domains, providing further insights into autonomic nervous system function (Table 3). In the **time domain**, COVID-19 and CVD patients exhibited significantly lower meanNN and SDNN values compared to healthy volunteers, indicating reduced overall HRV in the patient groups. RMSSD and pNN50, both indicators of short-term variability and parasympathetic activity, were also lower in COVID-19 and CVD patients, with the COVID-19 group showing the most pronounced reduction. This suggests impaired parasympathetic function in both patient groups, especially in COVID-19 patients.

In the **frequency domain**, the total power was higher in healthy volunteers, while the percentage of low-frequency (LF) power was elevated in both COVID-19 and CVD patients, reflecting increased sympathetic activity. High-frequency (HF) power, indicative of parasympathetic tone, was significantly reduced in the COVID-19 group compared to both CVD and healthy subjects. Although the LF/HF ratio, which assesses sympatho-vagal balance, did not significantly differ between groups, the changes in LF and HF percentages suggest autonomic imbalance in COVID-19 and CVD patients.

For the **regularity domain**, Sample Entropy (SampEn) was lower in COVID-19 patients compared to healthy controls, indicating reduced HRV complexity. The nonlinearity measure probAgr was significantly reduced in both COVID-19 and CVD groups, suggesting diminished autonomic flexibility in the patient groups relative to healthy volunteers.

In the **nonlinear domain**, both SD1 and SD2, representing short-term and overall variability, were significantly lower in COVID-19 and CVD patients compared to healthy individuals. The SD1/SD2 ratio was notably lower and the SD2/SD1 ratio was higher in healthy volunteers than COVID and CVD groups, highlighting a more balanced autonomic response in the control group compared to the dysregulation observed in the patient populations.

**Table 3:** HRV parameters for the COVID-19, CVD, and healthy volunteer groups. There is significant autonomic dysfunction in both COVID-19 and CVD patients, with reduced HRV and parasympathetic activity, and increased sympathetic dominance, particularly in the COVID-19 group. Numbers represent the median and interquartile range (IQR) values. * one way ANOVA p-value < 0.05 compared to the healthy volunteers; ** *p* < 0.001. + *p* < 0.05 between COVID-19 and CVD.

Domain	HRV parameter	COVID-19	Healthy volunteers	CVD control	*p*-value
Time	MeanNN (s)	0.72 ± 0.16**	0.92 ± 0.1	0.75 ± 0.22**	1.35E^− 6^
	SDNN (s)	0.02 ± 0.02**	0.06 ± 0.03	0.03 ± 0.03**	3.2E^− 4^
	RMSSD (s)	0.02 ± 0.02**^+^	0.05 ± 0.03	0.03 ± 0.02*	0.08
	pNN50 (%)	0.79 ± 11.75**^+^	20.99 ± 38.3	5.98 ± 17.02*	5.09E^− 4^
Frequency	VLF (%)	37.25 ± 28.76	38.69 ± 26.38	27.84 ± 31.98	0.15
	LF (%)	18.47 ± 18.18*^+^	23.30 ± 11.79	19.08 ± 13.92*	8.7E^− 5^
	HF (%)	13.69 ± 25.80^*+^	22.91 ± 21.86	18.87 ± 24.45	0.02
	LF/HF (n.u.)	1.038 ± 1.54	1.03 ± 0.78	0.84 ± 1.08	0.065
Regularity	SampEn (a.u.)	1.51 ± 0.79*^+^	1.67 ± 0.3	1.64 ± 0.50	0.036
	probAgr (a.u.)	0.17 ± 0.28**	0.40 ± 0.07	0.18 ± 0.21**	4.2E^− 15^
Nonlinear	SD1 (s)	0.04 ± 0.04**	0.08 ± 0.05	0.05 ± 0.04*	0.06
	SD2 (s)	0.08 ± 0.07**	0.17 ± 0.1	0.11 ± 0.1**	4.4E^− 5^
	SD1/SD2 (n.u.)	0.57 ± 0.35	0.39 ± 0.25	0.59 ± 0.29*	0.07
	SD2/SD1 (n.u.)	1.76 ± 1.54*	2.61 ± 1.31	1.73 ± 1.08*	0.05

### HRV analysis within COVID-19 patients across demographics

Within the COVID-19 group, significant differences in HRV parameters were observed across age, gender, race, and BMI (Table 4). Adult patients (≤ 65 years) displayed significantly lower MeanNN and SDNN values, indicating a more reduced HRV compared to older adult patients (> 65). Males had slightly higher MeanNN and SDNN values compared to females, suggesting marginally better autonomic function in men. White patients showed higher MeanNN and SDNN values than non-white patients, reflecting higher heart rate variability and parasympathetic dominance. Unexpectedly, no significant differences were found in values of HRV parameters between the BMI ≤ 30 and BMI > 30 subgroups. However, patients with BMI ≤ 30 had slightly higher MeanNN and hence lower heart rate, increased pNN50, and decreased LF/HF percentages compared to those with BMI > 30. Overall, the data within the COVID-19 group suggest that demographic factors such as age, gender, race, and BMI play a critical role in the autonomic response to COVID-19, with younger patients, females, non-white individuals, and those with higher BMI exhibiting more pronounced autonomic dysfunction.

**Table 4:** HRV parameters comparison for different subgroups of the COVID-19 population. Values are expressed as median ± IQR. * indicates a t-test p-value < 0.05 between the corresponding subgroups.

HRV parameters	≤ 65yr	> 65yr	Male	Female	White	Non-white	BMI ≤ 30	BMI > 30
MeanNN (s)	0.65 ± 0.19*	0.8 ± 0.17	0.76 ± 0.18	0.71 ± 0.16	0.76 ± 0.16*	0.63 ± 0.19	0.75 ± 0.21	0.71 ± 0.18
SDNN (s)	0.02 ± 0.02*	0.04 ± 0.04	0.03 ± 0.02	0.02 ± 0.025	0.03 ± 0.04*	0.02 ± 0.02	0.02 ± 0.05	0.02 ± 0.02
RMSSD (s)	0.02 ± 0.01*	0.03 ± 0.07	0.02 ± 0.04	0.023 ± 0.01	0.02 ± 0.04	0.02 ± 0.01	0.02 ± 0.07	0.02 ± 0.01
pNN50 (%)	0.0 ± 2.83*	5.82 ± 35.7	0.67 ± 2.52	0.9 ± 7.26	2.24 ± 21.53	0.01 ± 1.07	0.67 ± 35.87	0.64 ± 5.81
VLF (%)	41.51 ± 23.18	30.39 ± 34.78	39.36 ± 51.53	34.40 ± 20.99	36.66 ± 29.56	41.46 ± 22.08	30.80 ± 25.02	41.62 ± 23.92
LF (%)	9.28 ± 17.7*	20.80 ± 13.58	18.32 ± 25.91	18.62± 19.76	19.02 ± 19.17*	8.84 ± 14.59	21.29 ± 17.36	14.27 ± 14.4
HF (%)	6.50 ± 14.95*	21.58 ± 24.08	17.03 ± 3413	12.93 ± 20.78	17.03 ± 27.45*	7.29 ± 10.48	12.93 ± 27.74	13.58 ± 18.07
LF/HF [n.u.]	1.47 ± 1.42	0.88 ± 0.99	1.04 ± 0.88	1.0 ± 2.36	1.0 ± 1.57	1.59 ± 1.29	1.0 ± 1.77	1.13 ± 1.45
SampEn [a.u.]	1.16 ± 0.75	1.43 ± 0.65	1.04 ± 0.75	1.47 ± 0.66	1.50 ± 0.75	1.83 ± 0.97	1.43 ± 0.7	1.58 ± 0.85
probAgr [a.u.]	0.13 ± 0.24*	0.22 ± 0.20	0.17 ± 0.26	0.16 ± 0.24	1 ± 0.21	0.08 ± 0.16	0.17 ± 0.31	0.16 ± 0.2
SD1 (s)	0.03 ± 0.02*	0.05 ± 0.12	0.04 ± 0.06	0.04 ± 0.03	0.04 ± 0.06	0.03 ± 0.01	0.04 ± 0.13	0.04 ± 0.03
SD2 (s)	0.05 ± 0.06*	0.11 ± 0.13	0.09 ± 0.07	0.096 ± 0.09	0.1 ± 0.13*	0.05 ± 0.05	0.07 ±0.14	0.08 ± 0.06
SD1/SD2 [n.u.]	0.52 ± 0.35	0.59 ± 0.0.37	0.57 ± 0.38	0.57 ± 0.32	0.57 ± 0.36	0.47 ± 0.49	0.63 ± 0.45	0.46 ± 0.24
SD2/SD1 [n.u.]	1.99 ± 1.58	1.76 ± 1.25	1.77 ± 1.36	1.77 ± 1.18	1.79 ± 1.35	2.25 ± 1.53	1.60 ± 1.56	2.22 ± 1.07

### Age-related differences in HRV parameters

Next, we investigated the HRV parameters for adults (age ≤ 65 years) vs. senior adults (age > 65 years) for the three study populations (Table 5). There were only two subjects in the healthy volunteer group of age > 65 years. Therefore, we did not include a healthy age > 65 year group in this statistical analysis. For the ≤ 65 year age group, significant differences were observed in nearly all time-domain parameters, with COVID-19 patients showing reduced MeanNN, SDNN, RMSSD, and pNN50 compared to healthy volunteers, indicating reduced HRV. Additionally, significant differences were found in most frequency domain parameters (LF and HF) and nonlinear parameters (SD1, SD2) between COVID-19 patients and healthy volunteers. The CVD group displayed similar trends, showing impaired HRV compared to healthy volunteers in the same parameters. However, a notable distinction was that LF/HF was larger in COVID-19 patients vs. healthy volunteers and CVD patients, suggesting a shift in sympatho-vagal balance in this group. Interestingly, despite significant age differences between COVID-19 and CVD patients in the ≤ 65 group in MeanNN and pNN50, no such differences were observed in HRV parameters for the > 65 age group although SampEn was significantly lower in COVID-19 patients in this age group. This indicates that there is a reduced parasympathetic activity and sympathetic dominance in patients with COVID-19 and CVD patients than Healthy volunteers for ≤ 65 age group. Even though, there are significant differences in MeanNN (or heart rate) between COVID-19 and CVD patients for ≤ 65 age group, but in older patients (> 65 years), both groups exhibit similar HRV profiles.

**Table 5:** Age-related differences in HRV parameters. Values are expressed as median ± IQR. * indicates a t-test p-value < 0.05 compared to the healthy volunteers; ** indicates a t-test p-value < 0.001. + indicates a t-test p-value < 0.05 between COVID-19 and CVD control.

HRV parameters	Age ≤ 65 years			Age > 65 years		
	COVID-19	Healthy	CVD	COVID-19	Healthy	CVD
MeanNN (s)	0.65 ± 0.19**^+^	0.91 ± 0.09	0.76 ± 27*^+^	0.8 ± 0.17	-	0.73 ± 0.2
SDNN (s)	0.02 ± 0.02**	0.06 ± 0.03	0.03 ± 31.42**	0.04 ± 0.04	-	0.04 ± 0.04
RMSSD (s)	0.02 ± 0.01**	0.05 ± 0.03	0.02 ± 57.30*	0.03 ± 0.07	-	0.03 ± 0.04
pNN50 (%)	0.0 ± 2.83**	24.67 ± 40.60	2.28 ± 7.57^+^**	5.83 ± 35.68	-	13.1 ± 22.91
VLF (%)	41.51 ± 23.18	38.69 ± 25.42	35.08 ± 28.93	30.4 ± 34.78	-	19.66 ± 26.54
LF (%)	9.28 ± 17.70*	23.30 ± 10.16	16.72 ± 17.08*	20.81 ± 13.58	-	20.07 ± 13.98
HF (%)	6.5 ± 14.95**	22.61 ± 24.67	13.39 ± 12.29*	21.58 ± 24.08	-	22.84 ± 23.92
LF/HF [n.u.]	1.47 ± 1.42	0.98 ± 1.42	0.8 ± 1.35	0.88 ± 0.99	-	0.84 ± 1
SampEn [a.u.]	1.16 ± 0.75	1.73 ± 0.31	1.71 ± 0.47	1.43 ± 0.65^+^	-	1.57 ± 0.39
probAgr [a.u.]	0.13 ± 0.24**	0.4 ± 0.07	0.14 ± 0.18**	0.2 ± 0.2	-	0.19 ± 0.27
SD1 (s)	0.03 ± 0.02**	0.08 ± 0.05	0.04 ± 99.41**	0.05 ± 0.12	-	0.06 ± 0.06
SD2 (s)	0.05 ± 0.06**	0.19 ± 0.1	0.1 ± 66.52**	0.11 ± 0.13	-	0.13 ± 0.11
SD1/SD2 [n.u.]	0.52 ± 0.35	0.38 ± 0.25	0.51 ± 0.41	0.59 ± 0.37	-	0.6 ± 0.25
SD2/SD1 [n.u.]	1.99 ± 1.58	2.66 ± 1.19	2.04 ± 1.59	1.76 ± 1.26	-	1.66 ± 0.69

### HRV variations based on gender

When HRV parameters are compared between male and female study participants, in males, there are significant lower values in COVID-19 patients than healthy volunteers across several parameters, including MeanNN, SDNN, pNN50, and LF (Table 6) indicating impaired autonomic function compared to healthy individuals. The reduction in LF and HF percentages further emphasizes this autonomic dysfunction, with lower parasympathetic activity and sympathetic imbalance in COVID-19 male patients.

For females, similar trends were observed, with COVID-19 patients showing significant lower values in MeanNN, SDNN, and HF compared to healthy volunteers. Females also had significantly reduced parasympathetic activity, as evidenced by lower HF percentages and SDNN values in COVID-19 patients. Additionally, nonlinear parameters such as SD1, SD2 and probAgr showed significant lower valuers in COVID-19 vs. healthy females, indicating diminished HR complexity and nonlinearity in the COVID-19 group. Interestingly, both male and female CVD patients exhibited similar trends of reduced HRV compared to healthy volunteers, though the differences between COVID-19 and CVD patients were not statistically significant in either gender.

**Table 6:** Gender-based differences in HRV parameters. Values are expressed as median ± IQR. * indicates a t-test p-value < 0.05 compared to the healthy volunteers; ** indicates a t-test p-value < 0.001. + indicates a t-test p-value < 0.05 between COVID-19 and CVD control.

HRV parameters	Male			Female		
	COVID-19	Healthy	CVD	COVID-19	Healthy	CVD
MeanNN (s)	0.76 ± 0.21*	0.92 ± 0.10	0.73 ± 0.18*	0.71 ± 0.1**	0.92 ± 0.09	0.79 ± 0.1*
SDNN (s)	0.03 ± 0.05*	0.06 ± 0.03	0.04 ± 0.03*	0.02 ± 0.03*	0.06 ± 0.03	0.03 ± 0.03*
RMSSD (s)	0.02 ± 0.07	0.04 ± 0.03	0.03 ± 0.03	0.02 ± 0.03*	0.05 ± 0.03	0.03 ± 0.03*
pNN50 (%)	0.7 ± 35.87*	15.66 ± 43.52	7.17 ± 18.72	0.9 ± 26.51*	26.8 ± 25.51	4.7 ± 26.51*
VLF (%)	39.36 ± 25.39	44.06 ± 24.13	26.75 ± 25.53*	34.4 ± 24.27	32.45 ± 23.74	30.46 ± 24.74
LF (%)	18.32 ± 24.35*	22.73 ± 9.24	20.30 ± 15.53*	18.62 ± 16.84*	24.79 ± 14.67	15.82 ± 14.78*
HF (%)	17.03 ± 27.74	18.13 ± 22.27	20.86 ± 22.31	12.93 ± 25.26*	26.59 ± 21.92	14.91 ± 21.93*
LF/HF [n.u.]	1.04 ± 0.88	1.03 ± 1.39	0.86 ± 0.81	1 ± 2.36	1.02 ± 1.12	0.77 ± 1.17
SampEn [a.u.]	1.47 ± 0.75	1.64 ± 0.31	1.67 ± 0.40	1.67 ± 0.66	1.65 ± 0.34	1.62 ± 0.32
probAgr [a.u.]	0.18 ± 0.31**	0.37 ± 0.08	0.18 ± 0.21**	0.16 ± 0.24**	0.41 ± 0.04	0.18 ± 0.04**
SD1 (s)	0.04 ± 0.13	0.07 ± 0.06	0.05 ± 0.05**	0.04 ± 0.03*	0.08 ± 0.05	0.04 ± 0.05*
SD2 (s)	0.09 ± 0.14*	0.16 ± 122.70	0.11 ± 0.09**	0.07 ± 0.09*	0.19 ± 0.12	0.09 ± 0.11*
SD1/SD2 [n.u.]	0.57 ± 0.1	0.37 ± 0.37	0.6 ± 0.25**	0.57 ± 0.22	0.43 ± 0.28	0.58 ± 0.29
SD2/SD1 [n.u.]	1.77 ± 1.36	2.72 ± 0.84	1.74 ± 0.74	1.79 ± 1.18	2.34 ± 1.26	1.74 ± 1.11

### Race-based differences in HRV parameters

Another demographic aspect that we investigated in relations with COVID-19 was racial differences (Table 7). The HRV statistical analysis between White and non-White subjects revealed significant differences between COVID-19 patients and healthy volunteers in several key parameters. In White subjects, parameters such as MeanNN, SDNN, pNN50 and SD1 were significantly reduced in COVID-19 patients, indicating autonomic dysfunction with decreased HRV and reduced parasympathetic activity. These trends were similarly in the CVD group, where significant differences between CVD patients and healthy volunteers were noted across most HRV parameters. However, no HRV parameters except SampEn were significantly different between COVID-19 and CVD patients in the White subpopulation.

In non-White subjects, COVID-19 patients exhibited more pronounced autonomic dysfunction compared to their White counterparts, as reflected in lower values for key HRV parameters, such as MeanNN, SDNN, and HF power, as already shown in Table 4. Significant reductions in parameters such as MeanNN, pNN50, and probAgr were observed when comparing non-white COVID-19 patients to healthy volunteers, indicating diminished heart rate complexity and autonomic imbalance. Frequency-domain parameters like LF and HF also showed significant differences between non-White COVID-19 patients and healthy volunteers, although they did not differentiate between CVD and healthy individuals. Moreover, like in White subjects, nonlinear parameters from Poincaré plots (SD1/SD2, SD2/SD1) did not show significant differences across the study groups in non-White subjects, but a regularity measure (SampEn) for White and a time-domain parameter SDNN, and SD1 for non-white patients did highlight differences between COVID-19 and CVD patients. Overall, while both racial groups demonstrated reduced HRV in COVID-19 and CVD patients compared to healthy volunteers, non-White subjects experienced greater reduction in parasympathetic activity, particularly in frequency-domain and regularity parameters than CVD patients.

**Table 7:** Race-based differences in HRV parameters. Values are expressed as median ± IQR. * indicates a t-test p-value < 0.05 compared to the healthy volunteers; ** indicates a t-test p-value < 0.001. + indicates a t-test p-value < 0.05 between COVID-19 and CVD control.

HRV parameters	White			Non-white		
	COVID-19	Healthy	CVD	COVID-19	Healthy	CVD
MeanNN (s)	0.76 ± 0.16**	0.93 ± 0.14	0.76 ± 0.21*	0.63 ± 0.19*	0.89 ± 0.07	0.69 ± 0.3
SDNN (s)	0.03 ± 0.04*	0.05 ± 0.03	0.04 ± 0.03*	0.02 ± 0.02*^+^	0.06 ± 0.02	0.03 ± 0.03*
RMSSD (s)	0.02 ± 0.04*	0.04 ± 0.03	0.03 ± 0.03	0.02 ± 0.0*	0.05 ± 0.05	0.02 ± 0.02^*^
pNN50 (%)	2.24 ± 21.53*	18.62 ± 25.09	6.67 ± 22.33	0.01 ± 1.08*^+^	24.55 ± 48.89	2.98 ± 14.51*
VLF (%)	36.66 ± 29.56	38.69 ± 26.98	24.90 ± 40.84	41.46 ± 22.08	37.57 ± 25.46	31.65 ± 31.36
LF (%)	19.02 ± 19.17*	25.12 ± 16.31	18.88 ± 27.69*	8.84 ± 14.59*	22.06 ± 3.51	19.59 ± 14.21*
HF (%)	17.03 ± 27.45	18.23 ± 15.51	20.61 ± 43.54	7.29 ± 10.48*	31.21 ± 28.14	13.67 ± 23.02*
LF/HF [n.u.]	1.0 ± 1.57	1.31 ± 1.61	0.64 ± 1.34	1.59 ± 1.29	0.72 ± 0.69	0.86 ± 1.05
SampEn [a.u.]	1.50 ± 0.75^+^	1.63 ± 0.3	1.61 ± 0.52^+^	1.83 ± 1	1.67 ± 0.34	1.62 ± 0.39
probAgr [a.u.]	0.2 ± 0.21**	0.41 ± 0.05	0.17 ± 0.22**	0.08 ± 0.16*	0.37 ± 0.09	0.22 ± 0.2*
SD1 (s)	0.038 ± 0.06*	0.07 ± 0.05	0.05 ± 0.05	0.03 ± 0.01*^+^	0.09 ± 0.09	0.04 ± 0.03*
SD2 (s)	0.1 ± 0.13*	0.16 ± 0.12	0.11 ± 0.09*	0.05 ± 0.05*	0.18 ± 0.06	0.1 ± 0.11*
SD1/SD2 [n.u.]	0.57 ± 0.36	0.38 ± 0.14	0.63 ± 0.24*	0.47 ± 0.5	0.43 ± 0.38	0.50 ± 0.25
SD2/SD1 [n.u.]	1.79 ± 1.35	2.67 ± 0.81	1.59 ± 0.81*	2.25 ± 1.58	2.34 ± 1.53	1.99 ± 1.16

### Comparison of HRV parameters based on BMI across study groups

The most pronounced distinctions in HRV parameters among different patient groups were in the BMI ≤ 30 subgroup (Table 8). All time-domain parameters, except RMSSD, showed significant differences between COVID-19 patients and healthy volunteers. The frequency domain parameters, except for VLF, also revealed significant differences between these two groups. Moreover, LF, LF/HF were found significantly higher in COVID-19 vs. CVD patients suggesting increased sympathetic modulation whereas SampEn was lower in COVID-19 patients suggesting reduced heart rate complexity. The non-linear time domain parameters, excluding SD1 and SD1/SD2, were also significantly different between CVD controls and healthy volunteers. These findings suggest that the HRV parameters can effectively differentiate between COVID-19 and healthy individuals, as well as between CVD patients and healthy individuals, in the BMI ≤ 30 subgroup. In the BMI > 30 subgroup, lower or significantly lower values of all HRV parameters (except SD1/SD2) were found in COVID-19 and CVD patients vs. healthy individuals. However, no HRV parameters were significantly different between COVID-19 and CVD patients in this group, suggesting that HRV measures may be less effective at distinguishing between these two patient populations when BMI > 30. Overall, the findings suggest that HRV parameters are particularly useful for identifying autonomic dysfunction in COVID-19 patients with lower BMI, while distinctions between COVID-19 and CVD patients become less clear in patients with higher BMI.

**Table 8:** Comparison of HRV parameters based on BMI across study groups. Values are expressed as median ± IQR. * indicates a t-test p-value < 0.05 compared to the healthy volunteers; ** indicates a t-test p-value < 0.001. + indicates a t-test p-value < 0.05 between COVID-19 and CVD control.

HRV parameters	BMI ≤ 30			BMI > 30		
	COVID-19	Healthy	CVD control	COVID-19	Healthy	CVD control
MeanNN (s)	0.75 ± 0.21*^+^	0.88 ± 0.09	0.7 ± 0.2*	0.7 ± 0.18*	0.93 ± 0.1	0.75 ± 0.24*
SDNN (s)	0.02 ± 0.05*^+^	0.06 ± 0.03	0.03 ± 0.04*	0.02 ± 0.02*	0.06 ± 0.04	0.04 ± 0.04*
RMSSD (s)	0.02 ± 0.07	0.05 ± 0.03	0.03 ± 0.03	0.02 ± 0.01*	0.05 ± 0.04	0.03 ± 0.02*
pNN50(%)	0.67 ± 35.87*	20.99 ± 33.60	6.36 ± 24.97	0.64 ± 5.81*	32.02 ± 40.63	4.43 ± 11.77*
VLF(%)	30.80 ± 25.02	34.18 ± 25.13	21.35 ± 32.93	41.62 ± 23.92	43.88 ± 19.98	33.23 ± 37.62
LF(%)	21.29 ± 17.36^+^	22.73 ± 14.78	19.34 ± 13.48*	14.27 ± 14.4*	23.30 ± 10.17	19.42 ± 14.84
HF(%)	12.93 ± 27.74*	25.6 ± 23.27	19.85 ± 25.57	13.58 ± 18.07*	22.21 ± 19.43	14.44 ± 18.63*
LF/HF[n.u.]	1 ± 1.77^+^	0.84 ± 1.65	0.8 ± 0.63^+^	1.13 ± 1.45	1.29 ± 1.55	1.03 ± 1.33
SampEn[a.u.]	1.43 ± 0.7*^+^	1.65 ± 0.316	1.61 ± 0.4^+^	1.58 ± 0.852	1.63 ± 0.26	1.5 ± 0.88
probAgr[a.u.]	0.17 ± 0.31**	0.4 ± 0.06	0.2 ± 0.21**	0.16 ± 0.19**	0.4 ± 0.09	0.21 ± 0.21**
SD1(s)	0.04 ± 0.13	0.08 ± 0.06	0.05 ± 0.06	0.04 ± 0.03**	0.08 ± 0.07	0.04 ± 0.04*
SD2(s)	0.07 ± 0.14*	0.16 ± 0.1	0.1 ± 0.12*	0.07 ± 0.06**	0.21 ± 0.13	0.11 ± 0.13*
SD1/SD2[n.u.]	0.63 ± 0.45	0.5 ± 0.25	0.62 ± 0.26**	0.46 ± 0.24	0.37 ± 0.28	0.55 ± 0.32
SD2/SD1[n.u.]	1.6 ± 1.56*	2.42 ± 1.21	1.63 ± 0.71*	2.22 ± 1.07	2.67 ± 1.18	1.8 ± 1.47

### HRV analysis in age-matched groups

Since there is an age difference between groups, in order to compare HRV parameters in an age-matched group, we selected a subset of study population of 29 COVID-19 patients, 12 healthy control, 31 CVD control subject whose ages are between 30 and 70 years old (Table 9). Significant differences were observed between COVID-19 patients and healthy volunteers across several HRV parameters, particularly in the time and frequency domains. COVID-19 patients showed reduced HRV, reflected by lower MeanNN, SDNN, RMSSD, pNN50 and HF percentages, indicating impaired autonomic function and decreased parasympathetic activity compared to healthy individuals. Similarly, the CVD group exhibited reductions in HRV compared to healthy volunteers, and no differences between COVID-19 and CVD patients were observed except for pNN50.

**Table 9:** Age-matched comparison of HRV parameters. * indicates a one-way ANOVA p-value < 0.05 compared to the healthy volunteers; ** indicates a one-way ANOVA p-value < 0.001. + indicates a one-way ANOVA p-value < 0.05 between COVID-19 and CVD control.

Domain	HRV parameter	COVID-19	Healthy volunteers	CVD control	*p*-values (One way ANOVA)
Time	**MeanNN** (s)	0.68 ± 0.14**	0.92 ± 0.14	0.73 ± 0.28*	9.52E^− 6^
	**SDNN** (s)	0.02 ± 0.02*	0.04 ± 0.03	0.03 ± 0.02*	0.06
	**RMSSD** (s)	0.02 ± 0.01*	0.07 ± 0.02	0.02 ± 0.01	0.418
	**pNN50** (%)	0.37 ± 3.61*^+^	11.31 ± 20.87	3.24 ± 12.70	0.056
Frequency	**VLF** (%)	37.89 ± 24.93	39.24 ± 14.88	34.89 ± 28.33	0.56
	**LF** (%)	13.84 ± 18.01*	29.84 ± 19.85	19.59 ± 16.24*	1 E^− 4^
	**HF(%)**	7.30 ± 18.65*	18.21 ± 14.16	15.21 ± 16.26	0.059
	**LF/HF**[n.u.]	1.51 ± 1.55	1.34 ± 1.31	0.98 ± 1.26	0.256
Regularity	**SampEn**[a.u.]	1.57 ± 0.72*	1.75 ± 0.23	1.69 ± 0.55	0.34
	**probAgr**[a.u.]	0.14 ± 0.27**	0.41 ± 0.03	0.14 ± 0.18**	3.14E^− 8^
Nonlinear	**SD1**(s)	0.04 ± 0.0*	0.06 ± 0.04	0.04 ± 0.03	0.406
	**SD2**(s)	0.06 ± 0.06*	0.15 ± 0.1	0.1 ± 0.08*	0.02
	**SD1/SD2**[n.u.]	0.57 ± 0.34*	0.38 ± 0.09	0.53 ± 0.29*	0.049
	SD2/SD1[n.u.]	1.79 ± 1.36	2.66 ± 0.56	1.58 ± 0.77*	0.242

Additionally, nonlinear measures such as, SD1, SD2 and SD1/SD2 were significantly different between COVID-19 patients and healthy volunteers, indicating altered heart rate complexity in COVID-19 patients (Table 9). There was also a significant difference in the complexity measure SampEn across COVID-19 and healthy groups but not the CVD group, suggesting that HRV complexity is also affected along with HRV in the age-matched population. Overall, HRV reductions in COVID-19 and CVD patients indicate significant autonomic imbalance, with COVID-19 patients displaying more prominent autonomic dysfunction in comparison to healthy controls.

## Discussion

The primary objective of this study was to assess autonomic function in hospitalized COVID-19 patients using heart rate variability (HRV) analysis and to compare these results with those from healthy volunteers and patients with cardiovascular disease (CVD). Our comprehensive analysis, which included 14 HRV parameters covering time, frequency, and nonlinear domains, provides new insights into the autonomic nervous system’s response to acute SARS-CoV-2 infection. The findings demonstrate a consistent reduction in HRV among COVID-19 patients, indicating impaired autonomic regulation. Specifically, time-domain parameters such as SDNN and RMSSD were significantly lower in COVID-19 patients compared to healthy controls, reflecting reduced parasympathetic activity similar as observed before including in pediatric patients^1^. These results are consistent with the increased sympathetic tone and decreased heart rate complexity observed across nonlinear measures such as SampEn and SD1/SD2^1^.

Our findings align with previous studies highlighting autonomic dysfunction in COVID-19 patients. For instance, Kaliyaperumal et al. reported similar decreases in HRV metrics and emphasized the importance of early detection of autonomic imbalances to improve clinical outcomes^3^. Additionally, the observed increase in hypertension incidence during and after the pandemic, as noted by Trimarco et al., emphasizes the broader cardiovascular impact of COVID-19 and our findings on autonomic dysregulation^4^. Liviero et al. further support our results with their long-term follow-up study, which demonstrated persistent autonomic dysfunction even in healthcare workers with mild COVID-19, although gradual recovery was observed over time^6^. This underlines the necessity for continued monitoring and potential interventions to mitigate long-term effects^6^.

Our study uniquely explored the influence of demographic factors on autonomic function. Age was a critical determinant, with older adults showing HRV profiles similar to those of CVD patients, suggesting that age-related autonomic decline might worsen COVID-19’s impact^7^. This observation is consistent with Rigo et al.‘s findings, which identified long-term autonomic syndrome in a significant proportion of hospitalized patients, emphasizing the need for rehabilitative strategies^7^. Gender differences were also evident, with males experiencing more pronounced HRV reductions than females. Moreover, racial disparities emerged, with non-white COVID-19 patients showing more severe autonomic dysfunction than their white counterparts, possibly due to genetic or socioeconomic factors^6^.

Our findings emphasize the complexity of autonomic regulation in COVID-19 patients and highlight the importance of personalized clinical management. The presence of tachycardia in some patients reflects significant autonomic dysfunction, which can resemble or contribute to conditions like postural orthostatic tachycardia syndrome (POTS) and other autonomic disorders, as discussed by Gomez-Moyano et al.^18^. This suggests a need for targeted diagnostic and therapeutic strategies to manage tachycardia and other arrhythmia conditions, aiming to improve long-term outcomes and effectively address these specific cardiovascular challenges in COVID-19 patients.

### Limitations

While our study provides valuable insights into the autonomic dysfunction associated with COVID-19, several limitations must be addressed. The variability in ECG data acquisition methods—hospital telemetry for COVID-19 and CVD patients versus dedicated equipment for healthy volunteers—could introduce measurement inconsistencies^5^. Additionally, age disparities between the control and patient groups may have influenced HRV comparisons, as aging significantly affects autonomic function. The limited sample size for certain demographic subgroups, particularly non-white individuals, also restricts the generalizability of our findings. Lastly, the observational nature of our study precludes establishing causality, emphasizing the need for longitudinal studies to clarify these associations further.

## Conclusions and future research

Our study demonstrates that COVID-19 patients experience significant HRV alterations, indicating autonomic dysfunction with increased sympathetic and reduced parasympathetic activity. HRV analysis is a valuable, non-invasive tool for identifying patients at higher risk of adverse outcomes and guiding personalized clinical management.

Future research will involve larger, age-matched cohorts to clarify the impact of aging on autonomic dysfunction in COVID-19. Additionally, exploring autonomic changes across varying severities of illness and recovery stages will offer insights into the progression of autonomic dysfunction. Expanding studies to more diverse populations will further explain the role of race in autonomic responses and support the development of customized treatment strategies.

## Data Availability

The datasets generated during and/or analyzed during the current study are available from the corresponding author at Pennsieve.io.

## References

[1] Delogu, A. B. et al. Autonomic cardiac function in children and adolescents with long COVID: A case-controlled study. *Eur. J. Pediatr.* **183**, 2375–2382. 10.1007/s00431-024-05503-9 (2024). 38446228 10.1007/s00431-024-05503-9 PMC11035407

[2] Guo, T. et al. Cardiovascular implications of fatal outcomes of patients with coronavirus disease 2019 (COVID-19). *JAMA Cardiol.* **5**, 811–818. 10.1001/jamacardio.2020.1017 (2020). 32219356 10.1001/jamacardio.2020.1017 PMC7101506

[3] Kaliyaperumal, D., Rk, K., Alagesan, M. & Ramalingam, S. Characterization of cardiac autonomic function in COVID-19 using heart rate variability: A hospital based preliminary observational study. *J. Basic. Clin. Physiol. Pharmacol.* **32**, 247–253. 10.1515/jbcpp-2020-0378 (2021). 33705614 10.1515/jbcpp-2020-0378

[4] Trimarco, V. et al. Incidence of new-onset hypertension before, during, and after the COVID-19 pandemic: A 7-year longitudinal cohort study in a large population. *BMC Med.* **22**, 127. 10.1186/s12916-024-03328-9 (2024). 38500180 10.1186/s12916-024-03328-9 PMC10949764

[5] Kleiger, R. E., Stein, P. K. & Bigger, J. T. Jr. Heart rate variability: Measurement and clinical utility. *Ann. Noninvasive Electrocardiol.* **10**, 88–101. 10.1111/j.1542-474X.2005.10101.x (2005). 15649244 10.1111/j.1542-474X.2005.10101.x PMC6932537

[6] Liviero, F. et al. Long term follow-up of heart rate variability in healthcare workers with mild COVID-19. *Front. Neurol.* **15**, 1403551. 10.3389/fneur.2024.1403551 (2024). 38827576 10.3389/fneur.2024.1403551 PMC11141692

[7] Rigo, S. et al. The long-COVID autonomic syndrome in hospitalized patients: A one-year prospective cohort study. *Eur. J. Intern. Med.* **120**, 38–45. 10.1016/j.ejim.2023.08.018 (2024). 37652756 10.1016/j.ejim.2023.08.018

[8] Junarta, J., Riley, J. M. & Pavri, B. B. Describing heart rate variability in patients with chronic atrial fibrillation during hospitalization for COVID-19. *J. Arrhythm.* **37**, 893–898. 10.1002/joa3.12569 (2021). 34386114 10.1002/joa3.12569 PMC8339086

[9] O’Driscoll, M. et al. Age-specific mortality and immunity patterns of SARS-CoV-2. *Nature* **590**, 140–145. 10.1038/s41586-020-2918-0 (2021). 33137809 10.1038/s41586-020-2918-0

[10] Zhou, F. et al. Clinical course and risk factors for mortality of adult inpatients with COVID-19 in Wuhan, China: A retrospective cohort study. *Lancet* **395**, 1054–1062. 10.1016/S0140-6736(20)30566-3 (2020). 32171076 10.1016/S0140-6736(20)30566-3 PMC7270627

[11] Mol, M. B. A. et al. Heart-rate-variability (HRV), predicts outcomes in COVID-19. *PLoS One* **16**, e0258841. 10.1371/journal.pone.0258841 (2021). 34710127 10.1371/journal.pone.0258841 PMC8553073

[12] Aragon-Benedi, C. et al. Is the heart rate variability monitoring using the analgesia nociception index a predictor of illness severity and mortality in critically ill patients with COVID-19? A pilot study. *PLoS One* **16**, e0249128. 10.1371/journal.pone.0249128 (2021). 33760875 10.1371/journal.pone.0249128 PMC7990300

[13] Heart rate variability. Standards of measurement, physiological interpretation, and clinical use. Task Force of the European Society of Cardiology and the North American Society of Pacing and Electrophysiology. *Eur. Heart J.* **17**, 354–381 (1996). 8737210

[14] Richman, J. S. & Moorman, J. R. Physiological time-series analysis using approximate entropy and sample entropy. *Am. J. Physiol. Heart Circ. Physiol.* **278**, H2039–2049. 10.1152/ajpheart.2000.278.6.H2039 (2000). 10843903 10.1152/ajpheart.2000.278.6.H2039

[15] Fishman, M. et al. A method for analyzing temporal patterns of variability of a time series from Poincare plots. *J. Appl. Physiol. (1985)* **113**, 297–306. 10.1152/japplphysiol.01377.2010 (2012). 22556398 10.1152/japplphysiol.01377.2010 PMC3404703

[16] Shaffer, F. & Ginsberg, J. P. An overview of heart rate variability metrics and norms. *Front. Public. Health* **5**, 258. 10.3389/fpubh.2017.00258 (2017). 29034226 10.3389/fpubh.2017.00258 PMC5624990

[17] Aktaruzzaman, M. & Sassi, R. Parametric estimation of sample entropy in heart rate variability analysis. *Biomed. Signal Process. Control* **14**, 141–147. 10.1016/j.bspc.2014.07.011 (2014).

[18] Gomez-Moyano, E. et al. Postural orthostatic tachycardia syndrome and other related dysautonomic disorders after SARS-CoV-2 infection and after COVID-19 messenger RNA vaccination. *Front. Neurol.* **14**, 1221518. 10.3389/fneur.2023.1221518 (2023). 37654428 10.3389/fneur.2023.1221518 PMC10467287

